# l-Carnitine Mitigates Trazadone Induced Rat Cardiotoxicity Mediated via Modulation of Autophagy and Oxidative Stress

**DOI:** 10.1007/s12012-022-09759-1

**Published:** 2022-07-04

**Authors:** Naglaa F. Khedr, Ola A. El-Feky, Rehab H. Werida

**Affiliations:** 1 Department of Biochemistry, Faculty of Pharmacy, Tanta University, Al-Baher Street, Medical Campus, Tanta, 31527 Egypt; 2 Clinical Pharmacy & Pharmacy Practice Department, Faculty of Pharmacy, Damanhur University, El-Bahiara, Egypt

**Keywords:** Autophagy, Cardiotoxicity, l-carnitine, Oxidative stress, Trazodone, TNF-α

## Abstract

Trazodone (TRZ) is an antidepressant drug which widely used to treat insomnia, but it has a cardiotoxic effect which considered one of the TRZ limitations. The aim of this study was to investigate the protective role of l-carnitine in rats against TRZ-induced cardiotoxicity, as well as to look into the molecular mechanisms underlying its cardioprotective effects via autophagy-mediated cell death and oxidative stress. Male albino rats were randomized into four experimental groups (*n* = 8): normal control, TRZ group (TRZ, 20 mg/kg/day), l-carnitine group (LC, 200 mg/kg/day), and Co-treated group (l-carnitine and TRZ). All treatments were administered via oral gavage for 4 weeks. Cardiac enzymes (AST & CK-MB) and serum cardiac troponin T(cTnI) were assessed. Oxidative stress biomarkers in heart tissue (malondialdehyde; MDA, total thiol, and catalase activity) were measured. Autophagy related-genes (ATG-5 and Beclin-1), P62, and TNF-α were quantified. AST and CK-MB and cTnI significantly (*p* < 0.001) were increased with enhanced autophagy as well as severe histopathological changes which were manifested as scattered chronic inflammatory cells with focal fragmentation of myocardial fibers and loss of nuclei in TRZ-treated group. However, daily administration of l-carnitine (200 mg/kg) for 28 days completely reversed TRZ-induced the increased cardiac enzymes, autophagy, and myocardial inflammatory processes to the normal values. TRZ administration might have the potential to cause cardiotoxic effects that can be treated with l-carnitine administration.

## Introduction

Depression is a mood illness marked by feelings of melancholy, worry, insomnia, and suicide ideation. Trazodone (TRZ) is a serotonin receptor type 2 (5-HT2) antagonist and reuptake inhibitor that has been clinically approved since 2001. Despite the fact that TRZ is a pharmacologically approved treatment for insomnia, it has a long list of documented side effects, including headaches, dizziness, hypotension, syncope, and feeling off balance problems [1]. Also, previous studies reported cardiovascular adverse effects such as electrocardiogram (ECG) irregularities (prolonged PR interval & long QT syndrome), postural hypotension, and cardiac arrhythmias were induced at therapeutic and even subtherapeutic doses of TRZ [2]. However, the molecular pathogenesis and mechanisms by which TRZ exert its cardiac side effects still not fully understood.

One of the most prevalent mechanisms of drug-induced cardiotoxicity is the generation of reactive oxygen species (ROS) and free radicals. The production of reactive oxygen species (ROS) regulates the expression of many cytokines, including tumor necrosis factor (TNF-α), which serves as an essential inflammatory biomarker for cardiotoxicity [3, 4] Therefore, the inhibition of TNF-α through the administration of cardioprotective agents could have a potential therapeutic role.

Autophagy is one of the most recently discovered main cell death mechanisms, since it begins with the destruction and recycling of proteins and organelles, which are subsequently sequestered into double-membrane vesicles known as autophagosomes [5, 6]. Recent studies reported more than 30 autophagy-related (ATG) genes that stimulate autophagy through coding proteins essential for induction, maturation, and recycling of autophagosomes [7, 8] Furthermore, autophagy has been found to play a critical role in cardiac homeostasis mechanisms. The inhibition of autophagy gene 5 (ATG5) resulting in myocardial dysfunction, as well as inactivation of disruption of ATG6 (also known as Beclin1) accelerating heart failure in cardiomyopathic mice [3, 9]. Additionally in heart failure patients, autophagic myocyte death was observed. Cardiomyocytes are differentiated cells that lose their proliferative potential as a result of lethal injury such as necrosis, apoptosis, or autophagy, which causes heart malfunction and worsens cardiac failure [7].

l-carnitine (LC) (β-hydroxy-γ-N-trimethylaminobutyric acid) is required for the transport of long-chain fatty acids into mitochondrial matrix to start β-oxidation of fatty acids which is the primary energy source in the myocardium[10]. Previous studies demonstrated that LC has antioxidant, anti-inflammatory, and antiapoptotic properties which could be of value in protection against cardiotoxicity [11, 12]. The purpose of this study was to investigate the molecular mechanisms that explain l-carnitine's protection against TRZ-induced cardiotoxicity by examining its effects on autophagy, inflammation, and oxidative stress.

## Materials and Methods

### Drugs and Chemicals

Trazadone hydrochloride (TRZ) Trittico® (EIPICO, Tenth of Ramadan City—1^st^ Industrial Zone B1, Egypt). l-carnitine was purchased from Sigma-Aldrich, USA. Drugs were prepared in distilled water.

### Experimental Animals and Design

Male Albino rats weighing 150–200 g at 12 weeks of age were purchased from the animal house of Giza Institute of Ophthalmology, Cairo, Egypt. The animals were kept at12 h light–dark cycle and 25 ± 2 °C temperature. All the animals were allowed free access to diet and tap water. After a week of adaptation, rats were divided randomly and equally into four groups (*n* = 8) as follows:

Control group: Rats received vehicle (distilled water) orally for 28 days [13].
TRZ group: Rats received oral dose of TRZ (20 mg/kg BW/day) for 28 days [13]. TRZ at 20 mg/kg/day induces cardiotoxicity in rats, hence this dose was chosen based on past researches. TRZ causes a reduction in heart rate, a longer PR interval, and smaller QRS and T amplitudes. In addition, serum AST and cTn-T levels were considerably higher than in the control group [1, 13].
LC (lcarnitine) group: Rats received oral dose of l-carnitine (200 mg/kg BW/day) for 28 days. Several studies found that different dosages of LC, such as 150 and 300 mg/kg BW/day, had a protective impact on cardiomyocytes. Cardiotoxicity pathogenic alterations such necrotic muscle fibers and large inflammatory cellular infiltrations were reduced by LC [14, 15].
LC + TRZ (l-carnitine and TRZ) co-treated group: Rats received TRZ (20 mg/kg/day) along with l-carnitine (200 mg/kg/day) for 28 days via oral gavage.

At the end of the experiment (after 4 weeks), rats were kept fasted overnight and weighed. Then, rats were sacrificed by cervical dislocation under ether anesthesia. Aliquots of serum samples were collected and separated using cooling centrifuge at × 708 g (Laborzentrifugen 3-3OK, Sigma, Germany). Then, serum was stored at − 80 °C for biochemical analysis. Fresh hearts were dissected after scarification of rats, washed with normal saline, then dried, and weighed. Heart was cut into small portions. One portion was fixed in 10% formalin for histopathological examination, while the other portions were kept at − 80 °C for biochemical analysis and qRT-PCR measurements.

### Determination of Cardiac /Body Weight Ratio

Cardiac/body weight ratio was calculated as heart weight(g)/final body weight (fBW) (g) × 100 [15].

### Determination of Serum Cardiac Function Biomarkers

Serum levels of creatine kinase-MB (CK-MB) and aspartate aminotransferase (AST) were assayed spectrophotometrically using purchased kits from Biotecnica Instruments S.p.A, Rome, Italy. All procedures were performed according to manufacturer protocol. CK-MB was expressed as U/L and AST as IU/L. The detection limit for CK-MB was 2–2000 U/L and 4–700 IU/L for AST. Cardiac troponin T (cTnI) was determined with sandwich enzyme-linked immunosorbent assay (ELISA) technique using rat cTn-I ELISA kit (Sun-Red Biotechnology Company®, China) according to the manufacturer protocol. Detection limit for cTnI was 4–1000 ng/L.

### Preparation of Heart Tissue Homogenate

Heart tissue homogenate was prepared by homogenization of 0.2 g of cardiac tissue in two mL phosphate buffered saline (pH 7.4, (1:10) w:v ratio) using Polytron homogenizer (PT 3100, Switzerland) at × 800 g for 20 min at 4˚C and the supernatant was separated immediately and stored at – 80 °C until future analysis [16].

### Determination of P62 in Heart Tissue

The autophagy protein P62 in heart tissue homogenate was determined with double-antibody sandwich enzyme-linked immunosorbent assay (ELISA) using ELISA kit (Sun-Red Biotechnology Company®, China) according to the manufacturer protocol. The detection limit was 15–2000 pg/mL.

### Determination of Oxidative Stress Biomarkers

The lipid peroxidation biomarker, malondialdehyde (MDA), was determined using MDA assay kit purchased from (Spectrum-Diagnostics®). The assay is based on the interaction of malondialdehyde (MDA) with thiobarbituric acid (TBA), which results in the formation of an MDA-TBA2 adduct with a strong absorption at 532 nm. MDA was expressed (nmol/g tissue) using a molar extinction coefficient for MDA of 1.56 × 10^5^ M^−1^ cm^−1^[17]. Catalase (CAT, EC 1.11.1.6) activity was measured by monitoring H_2_O_2_ at 240 nm as previously described by Aebi [18]. The total thiol content of the heart was determined using Sedlak and Lindsay's technique [19]. The yellow dianion of 5-thio-2-nitrobenzoic acid (TNB) is formed when total thiol is treated with DTNB (5,5′-dithiobis-2-nitrobenzoate). The total thiol content was determined using a 13,600 M^−1^ cm^−1^ molar extinction coefficient and expressed as mmoL/g tissue.

### Gene Expression of Beclin-1, ATG-5 & TNF-α by Quantitative Real-Time Polymerase Chain Reaction (qRT-PCR)

Total RNA extraction kit (Bio E-Technology, China) was used for extraction of total RNA from heart tissue, purity and concentration of mRNA were estimated at A260/A280 nm using Nanodrop spectrophotometer (Analytik Jena, Italy). Complementary DNA (cDNA) was synthesized from extracted mRNA using cDNA synthesis kit (Origene™ Technologies, USA). The cDNA was amplified using SensiMix SYBR Master Mix (Origene™ Technologies, USA). Primer sequences were designed by Primer 3 plus Program (version 2.0) and purchased from Biosearch Technologies Co., (California, USA). Primer sequences of ATG-5: F (5′-GGCATGCTTCCCTAACTTGA-3′) & R (5′-CCCACCCATCCAAGAGTACA-3′), Beclin-1 (ATG-6): F (5`-TGGATCTGGACCAGGGGTCCTTGCG-3’) & R (5′-GTTTCGCCTGGGCTGTGGTAAGTAA-3`), TNF-α: F (5′-CTTCTCCTTCCT GAT CGT GG-3′) & R (5′-GCT GGT TATCTCTCAGCT CCA-3′) and GAPDH (Control Gene): F (5′-GAAGGT GAA GGTCGG AGT-3′) & R (5′-GAAGATGGT GATGGG ATT TC-3′). Each sample was analyzed by qRT-PCR system Pikoreal 5100 (Thermo Fisher Scientific Co., Finland), normalized to the level of the reference gene, and threshold cycle (*C*_t_) values were calculated. The relative gene expression level was calculated by using 2^−Δ*C*t^ formula [20].

### Histopathological Examination of Heart Tissue

Sections of cardiac tissue were fixed in 10% formalin for 24 h, then transferred to various grades of alcohol, cleaned in xylene, and finally embedded in paraffin to form blocks. Then sections were cut into 5-μm using microtome (Leica RM2135, Germany), deparaffinized and stained with hematoxylin, and eosin (H&E). Heart sections were examined by two expert histopathologists, who are blinded for treatment using a light microscope (Leica DM 500, Switzerland). Necrosis, apoptosis, and inflammatory cells were examined and scored as histopathological changes according to Joukar et al. [21]: (0) nil; (1) minimum (focal myocytes damage); (2) mild (small multifocal degeneration with slight degree of inflammatory process); (3) moderate (extensive myofibrillar degeneration and/or diffuse inflammatory process); (4) severe (necrosis with diffused inflammatory process).

### Immunohistochemical Assay for Autophagy Marker LC3II

Paraffin-embedded specimens were deparaffinized in xylene and dehydrated in graded alcohol. 3% hydrogen peroxide in phosphate buffered saline was used to inhibit endogenous peroxidase activity. After 20 min incubation with a protein-blocking solution consisting of PBS with 1.5% normal goat serum (DAKO, Glostrup, Denmark), sections were incubated overnight at 4 °C with the appropriate dilution of Rabbit monoclonal [EPR18709] to LC3BII—Autophagosome Marker (Abcam, UK) antibody. After that, the sections were treated for 30 min with the correct dilution of biotinylated anti-mouse IgG (Vectastain Elite Avidin–Biotin Complex kit, Vector Labs, Burlingame, CA). Stable 3,3′-diaminobenzidine tetrahydrochloride was used to observe immunocomplexes (Dojin, Kumamoto, Japan). The sections were washed in distilled water and counterstained with Mayer's hematoxylin for 10 s [22].

#### Statistical Analysis

Data are presented as mean ± SD and as a percent of change. Statistical analysis was performed with statistical package for social science (SPSS) software version 20 [23]. Statistical comparison among groups was performed by one-way analysis of variance (ANOVA) using Fisher’s least significant differences (LSD) for comparison between groups. Statistical significance was set at *p* < 0.05.

## Results

### Effect on Heart Weight

There was a non-significant difference between the studied groups in body weights, heart weight/fBW ratio after 4 weeks of treatment (Table 1). Whereas heart weights of LC + TRZ-treated groups showed significant decrease compared to control (*p* = 0.001), TRZ (*p* = 0.00), and LC (*p* = 0.025) treated groups.

**Table 1:** Effect of treatment on body weight and heart weight of rat groups

Parameter	Control	TRZ	LC	LC + TRZ	*P-ANOVA
iBW (g)	123.37 ± 13.34	146.75^a^ ± 15.37	135 ± 30.7	126.5 ± 22.77	0.159
fBW (g)	154.01 ± 19.41	170.79 ± 16.65	157.25 ± 34.19	146.25 ± 26.32	0.283
Heart weight (g)	0.71 ± 0.07	0.73 ± 0.06 ^c^* p* = 0.05	0.63 ± 0.14	0.52 ± 0.09 ^a^*p* = 0.001 ^b^*p* = 0.000 ^c^*p* = 0.025	0.001
Heart/fBW ratio %	0.46 ± 0.07	0.43 ± 0.07	0.43 ± 0.16	0.37 ± 0.12	0.439

Data were expressed as mean ± SD. *p* < 0.05 was set as significant

*TRZ (Trazadone)* 20 mg/kg/day, for 4 weeks, *LC (L-carnitine)* 200 mg/kg/day, for 4 weeks, *iBW* initial body weight (g), *fBW* final body weight (g)

^a^Significant vs. control

^b^Significant vs TRZ

^c^Significant vs LC

### Effect of Treatment on Cardiac Biomarkers and Autophagy Protein P62 Levels

As shown in Table 2, TRZ treatment significantly (*p* = 0.00) increased serum levels of CK-MB, AST, and cTnI compared to control. On the contrary, co-treatment with LC and TRZ significantly normalized serum levels of CK-MB (*p* = 0.00), AST (*p* = 0.049), and cTnI (*p* = 0.035) compared to TRZ group. Moreover, TRZ increased cardiac P62 level compared to control (*p* = 0.00), LC (*p* = 0.00), and LC + TRZ (*p* = 0.003) groups. Whereas co-administration of LC at dose of 200 mg/kg significantly (*p* = 0.003) modulated the effect of TRZ and attenuated the elevation of P62 compared to TRZ. However, LC only treated group showed normal level of cardiac biomarkers and reduced level of P62 compared to control (Table 2).

**Table 2:** Effect of treatment on cardiac biomarkers of studied rat groups

Parameter	Control	TRZ	LC	LC + TRZ	*p*-ANOVA
CK-MB (U/L)	149.25 ± 19.36	464.50 ± 50.86 ^a^*p* = 0.00 ^c^*p* = 0.00	128.00 ± 15.21 ^b^*p* = 0.00	272.25 ± 79.23 ^a^*p* = 0.00 ^b^*p* = 0.00 ^c^*p* = 0.00	0.000
AST (IU/L)	116.00 ± 31.27	346.20 ± 14.32 ^a^*p* = 0.00 ^c^*p* = 0.00	128.75 ± 18.16 ^b^*p* = 0.00	166.25 ± 58.82 ^a^*p* = 0.049 ^c^*p* = 0.00	0.000
cTn-I (ng/L)	238.30 ± 36.05	321.16 ± 17.11 ^a^*p* = 0.00 ^c^*p* = 0.000	256.46 ± 30.27 ^a^*p* = 0.002 ^b^*p* = 0.001	275.06 ± 43.63 ^a^*p* = 0.035 ^b^*p* = 0.001	0.000
P62 (pg/mL)	318.38 ± 31.67	664.15 ± 76.78 ^a^*p* = 0.00 ^c^*p* = 0.00	236.50 ± 30.75 ^a^*p* = 0.00 ^b^*p* = 0.00	410.74 ± 38.15 ^b^*p* = 0.003 ^c^*p* = 0.00	0.000

Data were expressed as mean ± SD. *p* < 0.05 was set as significant

*CK-MB* creatine kinase-myocardial band, *AST* aspartate transaminase, *cTn-I* cTroponine-TI, *TRZ (Trazadone)* 20 mg/kg/day, for 4 weeks, *LC (l-carnitine)* 200 mg/kg/day, for 4 weeks

^a^Significant vs. control

^b^Significant vs TRZ

^c^Significant vs LC

### Effect of Treatment on Cardiac Oxidative Stress Biomarkers

Oxidant and antioxidant status in rat heart were examined by measuring catalase activity, total thiol level, and the lipid peroxidation marker MDA (Table 3). TRZ induced cardiotoxicity and increased the oxidative stress in rat hearts. Total thiol (57.78 ± 16.02, *p* = 0.00) and catalase activity (8.35 ± 1.44, *p* = 0.049) were significantly decreased with an increase in MDA level (5.96 ± 1.41, *p* = 0.00) as compared to control, LC, and LC + TRZ treated groups. However, co-treatment with LC in LC + TRZ group significantly increased total thiol (129.63 ± 31.53, *p* = 0.00) and catalase activity (17.57 ± 5.15, *p* = 0.001) with decreased MDA level compared with TRZ group. LC exerted antioxidant effect and showed normal levels of total thiol and increased catalase activity compared to control **(**Table 3).

**Table 3:** Effect of treatment on cardiac oxidative stress biomarkers of studied rat groups

Parameter	Control	TRZ	LC	LC + TRZ	*p*-ANOVA
Total Thiol (mmoL/g tissue)	300.00 ± 43.66	57.78 ± 16.02 ^a^*p* = 0.00 ^c^*p* = 0.00	328.25 ± 68.29 ^a^*p* = 0.00 ^b^*p* = 0.00	129.63 ± 31.53 ^a^*p* = 0.00 ^b^*p* = 0.018 ^c^*p* = 0.00	0.000
MDA (nmol/g tissue)	1.98 ± 0.70	5.96 ± 1.41 ^a^*p* = 0.00 ^c^*p* = 0.00	2.50 ± 1.36 ^b^*p* = 0.00	2.79 ± 0.11 ^b^*p* = 0.00 ^c^*p* = 0.00	0.000
Catalase Activity (μmol/g tissue/min)	13.19 ± 4.17	8.35 ± 1.44 ^a^*p* = 0.049 ^c^*p* = 0.00	21.17 ± 6.5 ^a^*p* = 0.002 ^b^*p* = 0.007	17.57 ± 5.15 ^b^*p* = 0.001	0.000

Data were expressed as mean ± SD. *p* < 0.05 was set as significant

*TRZ (Trazadone)* 20 mg/kg/day for 4 weeks, *LC (l-carnitine)* 200 mg/kg/day for 4 weeks, *MDA* malondialdehyde

^a^Significant vs. control

^b^Significant vs. TRZ

^c^Significant vs LC

### Effect of Treatment on TNF-α and Autophagy Proteins Gene Expressions (Beclin1 and ATG5)

**Fig. 1:**
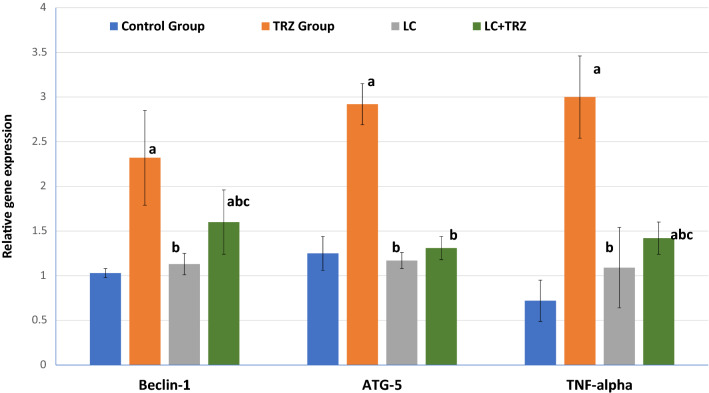
Relative gene expression of cardiac Beclin-1, ATG-5 and TNF-α. Data were expressed as mean ± SD. *p* < 0.05 was set as significant, ^a^significant *vs.* control, ^b^significant *vs* TRZ, ^c^significant *vs* LC. TRZ (Trazadone): 20 mg/kg/day, for 4 weeks; LC (l-carnitine): 200 mg/kg/day, for 4 weeks

By RT-PCR, we found that gene expression of Beclin1, ATG5 & TNF-α genes were significantly (*p* = 0.000) higher in heart of rats received TRZ for 4 weeks compared to control group (Fig. 1). Whereas the administration of l-carnitine at dose of 200 mg/kg significantly modulated the effect of TRZ and significantly decreased autophagy gene expression of Beclin1 (*p* = 0.007), ATG5 (*p* = 0.00), and the proinflammatory mediator TNF-α (*p* = 0.00) compared to TRZ treated group. However, LC only treated group showed significant (*p* = 0.00) decrease in Beclin1, ATG5, and TNF-α compared to TRZ with non-significant change compared with control group (Fig. 1).

### Effect of Treatment on Cardiac Histology

**Fig. 2:**
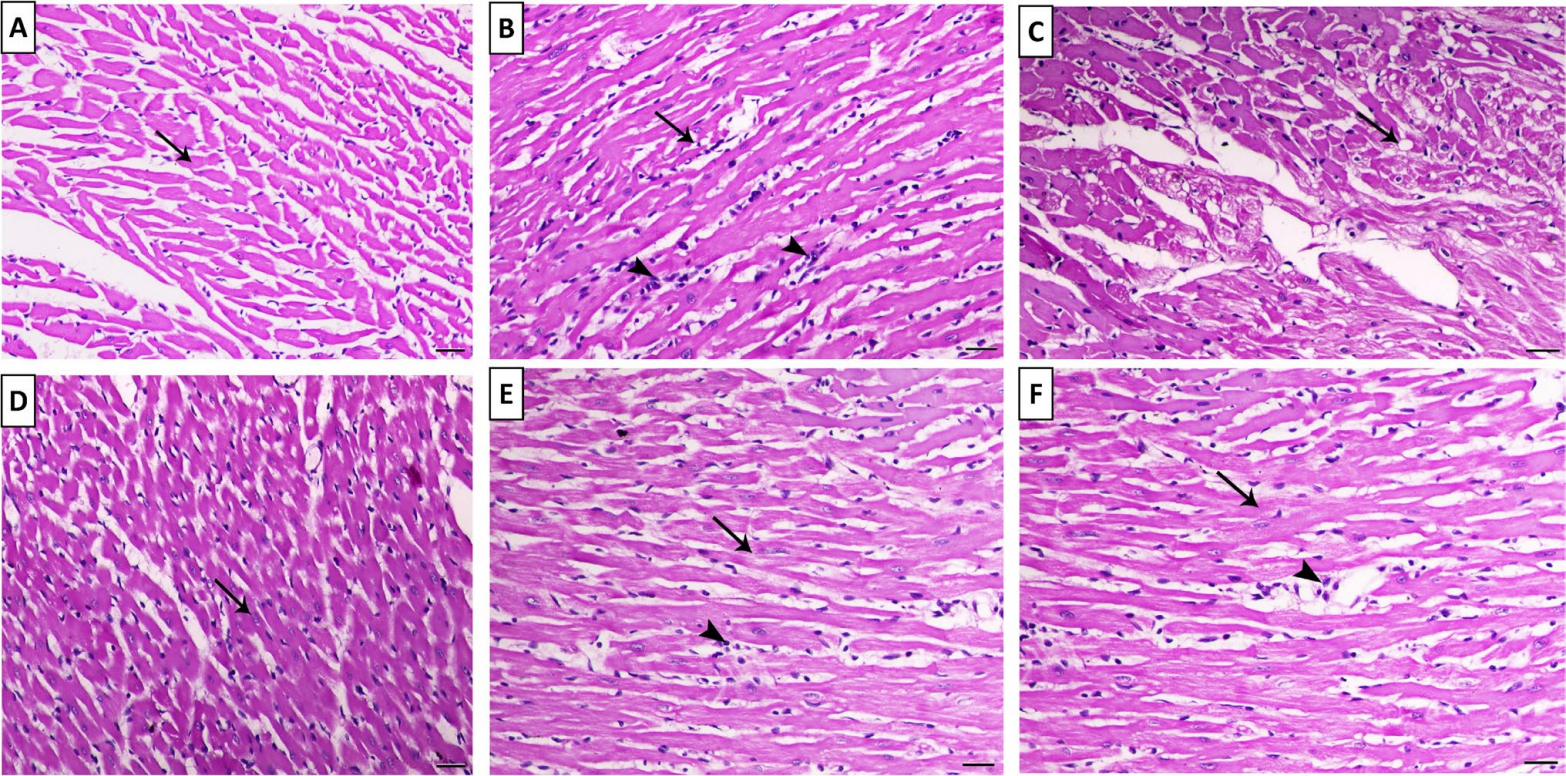
Photomicrographs of heart tissue (H&E, X100, bar = 50 µm). **a** Heart of control group showing normal branched myocardial fibers (arrow), **b** Heart of TRZ group showing myocarditis features associated with fragmentation of myocardial fibers (arrow)and infiltration of mononuclear cells mostly macrophages and lymphocytes (arrowheads), **c** Heart of TRZ group showing marked degree of myocardial necrosis and myolysis (arrow indicates fatty vacuolation within the bundles), **d** Heart of LC treated with rats showing normal cardiac muscle fibers with normal centrally located cigar-shaped nucleus (arrows), **e** Heart of rats treated with LC + TRZ showing mild degree of myolysis (arrow) associated with mild interstitial mononuclear inflammatory cells infiltration (arrowhead), **f** Heart of LC + TRZ-treated group showing focal myolysis (arrow) associated with mild interstitial mononuclear inflammatory cells infiltration (arrowhead). TRZ group; rats received oral dose of trazadone (20 mg/kg/day) for 28 days, LC (l-carnitine) group; rats received oral dose of l-carnitine (200 mg/kg/day) for 28 days, LC + TRZ (l-carnitine and TRZ) co-treated group; rats received oral dose of trazadone (20 mg/kg/day) along with oral dose of l-carnitine (200 mg/kg/day) for 28 days

In tissue sections from control rats, histological examination revealed a distinct morphology of cardiac cells with no signs of localized necrosis or inflammatory cell infiltration (Table 4 and Fig. 2a). On the contrary, the histopathological examination of heart tissue showed that TRZ treatment significantly increased the sarcoplasmic eosinophilia, vacuolation, necrosis, atrophy, and inflammation compared to the control and LC groups (Table 4 and Fig. 2b and c). Figure 2b and c shows that TRZ treatment resulted in myocarditis features that associated with fragmentation of myocardial fibers and infiltration of mononuclear cells mostly macrophages and lymphocyte and necrosis. However, treatment with LC + TRZ group showed mild degree of myolysis that was associated with mild interstitial mononuclear inflammatory cells infiltration (Fig. 2e, f). LC treatment in TRZ group also improved the increased sarcoplasmic eosinophilia, vacuolation, necrosis, atrophy, and inflammation compared with TRZ group (Table 4). Moreover, LC-treated groups showed normal cardiac muscle fibers with normal centrally located cigar-shaped nucleus (Fig. 2d).

**Table 4:** Semiquantitative scoring of cardiac lesions of studied rat groups

	Sarcoplasmic eosinophilia	Vacuolation	Necrosis	Atrophy	Inflammation
Control	−	−	−	−	−
LC	−	−	−	−	−
TRZ	+ + + +^ac^	+ + + +^ac^	+ + +^ac^	+ + + +^ac^	+ + + +^ac^
LC + TRZ	+^b^	+^b^	+^b^	+^b^	+^b^

Data were expressed as mean ± SD. *p* < 0.05 was set as significant

*TRZ (Trazadone)* 20 mg/kg/day, for 4 weeks, *LC (l-carnitine)* 200 mg/kg/day, for 4 weeks, *(−)* no alteration, *(*+*)* minimum (focal myocytes damage), *(*++*)* mild (small multifocal degeneration with slight degree of inflammatory process), *(*+++*)* moderate (extensive myofibrillar degeneration and/or diffuse inflammatory process), (++ ++) severe (necrosis with diffuse inflammatory process)

^a^Significant vs. control

^b^Significant vs. TRZ

^c^Significant vs. LC

### Effect of Treatment on LC3II Cardiac Expression

**Fig. 3:**
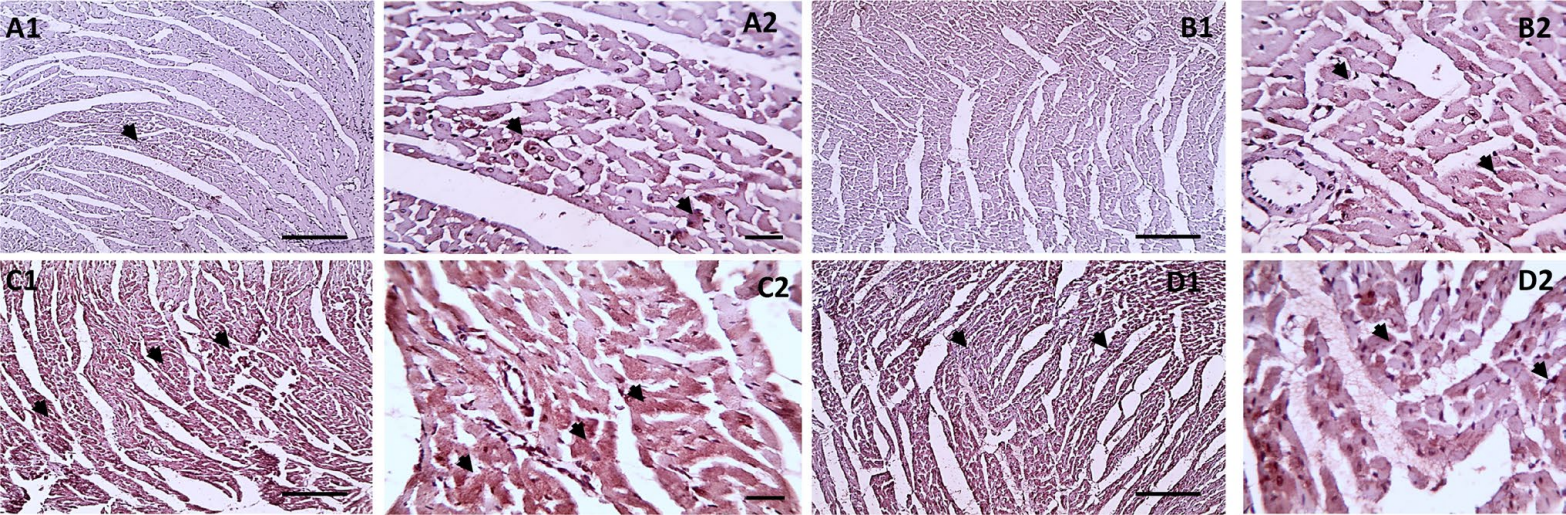
Microscopic pictures of immunostained cardiac sections against LC3II showing mild positive brown staining in muscle fibers in control group (a1 & a2). Heart of LC-treated group showing mild positive brown staining in cardiac fibers (b1 & b2). Cardiac sections from Trazadone (TRZ) group showing widespread strong positive brown LC3 expression in muscle fibers (arrowheads) (c1 & c2). Cardiac sections from TRZ + LC group showing markedly decreased positive brown staining (arrowheads) (d1 & d2). IHC counterstained with Mayer's hematoxylin. Low magnification (X: 100 bar 100) and high magnification (X: 400 bar 50)

**Fig. 4:**
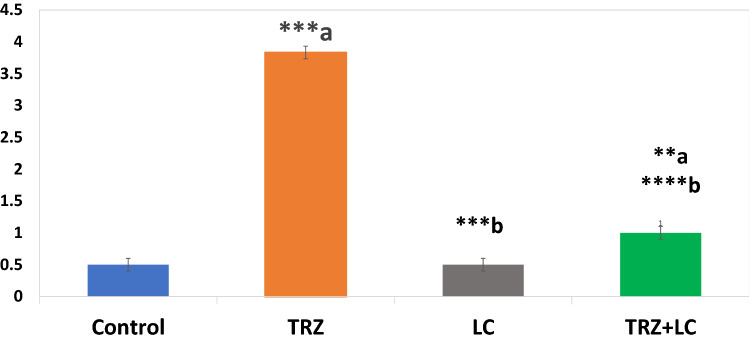
Showing Bars represented data of image analysis of LC3II immunoexpression in heart sections from all groups (mean ± SD). Scores of staining intensities as follows: (0–4) in 6 fields; 0 negative, 1 mild, 2 moderate, 3 strong, 4 very strong. ^a^significant vs. control, ^b^significant vs TRZ, TRZ (Trazadone): 20 mg/kg/day, for 4 weeks; LC (l-carnitine): 200 mg/kg/day, for 4 weeks

Figure 3 shows immunohistochemical detection of the LC3II-autophagosome protein expression in cardiac tissue slices. Control (Fig. 3a1 and a2) and LC (Fig. 3b1 and b2) groups cardiac sections reveal modest positive brown staining in muscle fibers. TRZ-treated group showing widespread strong positive brown LC3 expression in cardiac fibers indicating enhanced autophagy by trazadone toxicity (Fig. 3c1 and c2). Cardiac sections from the TRZ + LC group, on the other hand, showed much less positive brown staining (Fig. 3d1 and d2). Scoring of LC3II expression in cardiac sections was showed in Fig. 4 as follows: (0–4) in 6 fields 0 negative, 1 mild, 2 moderate, 3 strong, 4 very strong.

## Discussion

Cardiovascular side effects can arise as a result of acute or chronic pharmacological treatment, and they might affect the mechanical functioning of the myocardium and/or structure (e.g., morphological damage or loss of cellular/subcellular components of the heart or vasculature [24]. The cardiovascular adverse effects and pathological cardiovascular findings of TRZ were investigated and compared to l-carnitine combination with TRZ in the present study.

Trazodone, a tetracyclic SARI, has been linked to cardiovascular problems in multiple studies. In the heart, it has the potential to block calcium, potassium, and sodium channels. Trazodone can also cause drug-induced long QT syndrome, which can cause life-threatening cardiac arrhythmias in persons with mutations in cardiac ionic channel genes [25]. TRZ prolonged PR interval has also been linked to hypertrophy and other structural abnormalities like as cardiomyopathy [1].

Myocardial damage leads myocytes to release biomarkers like AST into the bloodstream, albeit it does not have total specificity to the myocardium. Also, CK-MB had a short half-life, indicating acute myocardial injury. In addition, cardiac troponin T, is one of the most important specific indicators of myocardial ischemic injury [26, 27]. The current investigation found that TRZ administration elevated serum CK-MB, AST, and cTnI levels, as well as a substantial increase in cardiac necrosis and myolysis in rat hearts. Previous studies support our findings that TRZ treatment induces myocardial ischemia [1, 28].

l-carnitine is a crucial component of energy metabolism and blood glucose control. l-carnitine helps transport long-chain fatty acids through the inner mitochondrial membrane, where they become a key substrate in energy generation and are degraded through beta-oxidation in muscle tissue and the heart's myocardium. Because the myocardium prefers to metabolize long-chain fatty acids for energy, l-carnitine becomes especially vital in the heart [29]. l-carnitine helps eliminate toxins from inside the mitochondria, mediates oxidative stress, inhibits fatty acid ester formation during ischemia events, and prevents cardiac cell death [30]. Herein, the dose of 200 mg/kg l-carnitine administered for 4 weeks significantly modulated TRZ cardiotoxicity; serum levels of CK-MB, AST, and cTnI levels were significantly decreased.

Clinical studies have shown, l-carnitine supplementation can enhance myocardial fat metabolism due to the increased demand for free fatty acids and their metabolites and thus creates a beneficial effect on myocardial function [31, 32]. Besides, therapeutic potentials of l-carnitine have been investigated in many rat models, for instances, in tilmicosin-induced cardiotoxicity [33], and imatinib-induced cardiotoxicity [34].

In the heart, reactive oxygen species are produced during normal cellular functions of mitochondria during oxidative phosphorylation as well as enzymatic reactions catalyzed by xanthine oxidase, NAD(P)H oxidases, and cytochrome P450 [35]. As the oxidative status of the cardiac tissue was assessed following TRZ treatment, a diminished catalase activity and increased MDA levels were reported, as well as a decrease in total thiol levels, when compared to the control, LC, and LC + TRZ groups. As a result, it's possible that the observed cardiac toxicity was accompanied with oxidative stress in cardiac tissue, which was alleviated by l-carnitine delivery. Also, in vitro studies showed that TRZ and its reactive metabolite caused oxidative stress by increasing MDA levels and depletion of glutathione [36]. l-carnitine, on the other hand, restored oxidative stress by lowering ROS and increasing endogenous antioxidant levels. In a dose-dependent manner, l-carnitine has radical scavenging capabilities and metal chelating activity [37].

In the TRZ-treated group, histological examination indicated a change in the shape of cardiac cells, as well as evidence of localized necrosis and inflammatory cell infiltration. Increased oxidative stress in cardiac tissue can lead to structural alterations like cardiomyopathy, cardiac hypertrophy, development of interstitial cardiac fibrosis, endothelial dysfunction, and contractile protein failure [38]. However, l-carnitine treatment successfully protected the heart as indicated by the improvement of the histopathological parameters.

During cellular stress, ATG5, a protein essential for the creation of the autophagy precursor, is cleaved by a cysteine protease and plays a key role in the initiation of autophagy. ATG12 is required for the development of autophagosomes. This protein undergoes ATP-dependent conjugation to ATG5, which is mediated by ATG7 and ATG10. Following that, the ATG12-ATG5 complex interacts with ATG16L1, generating an ATG12-ATG5-ATG16L1 conjugate that is required for effective Atg8/LC3 conjugation to phagophore membranes [39]. However, Beclin-1, which normally binds to Bcl-2 and inhibits autophagy, is competitively displaced, increasing the autophagic process under autophagy-inducing conditions [40]. P62 also increases aggresome formation and autophagy activation, as well as protecting cardiomyocytes from proteotoxic stress [41].

Following autophagy induction, the autophagy proteins ATG7, ATG3, and the ATG12-ATG5-ATG16L1 complex covalently conjugate MAP1LC3-I to phosphatidylethanolamine (PE) to generate MAP1LC3-II, which is then attracted to the inner and outer surface of autophagosomal membranes via PE. As a result of the development of MAP1LC3-positive puncta, autophagosomes can be easily detected immunohistochemistry in tissue sections [42]. Herein, the present study showed high expression of autophagosomal LC3II in cardiac tissues of TRZ-treated groups. However, LC treatment ameliorated this effect.

Cardiomyocytes, being postmitotic cells with high rates of energy consumption and an insatiable demand for ATP, have a plethora of mitochondria and are thus especially vulnerable to damaged or dysfunctional mitochondria. Cellular functions worsen as ROS accumulate, which is followed by autophagy disruption [43]. Herein, the present study showed that the expression of the Beclin1 and ATG5 genes, as well as the level of the p62 protein, were significantly higher in the hearts of rats given TRZ for 4 weeks compared to the control group. When compared to the TRZ-treated group, l-carnitine administration significantly reduced the effect of TRZ and reduced autophagy gene expression of Beclin1 and ATG5, as well as p62 protein levels. These effects could be explained by the increased oxidative stress situation caused by TRZ and controlled by l-carnitine 's antioxidant and scavenging abilities [13].

The current study showed increased gene expression of cardiac TNF-α following TRZ treatment. However, LC downregulated cardiac TNF-α gene expression. These results were in consistence with previous reports [37, 44, 45]. TNF-α is a pro-inflammatory cytokine produced by all cardiac cells in response to stress in order to initiate an inflammatory response. TNF-α binds to its receptor that have a death domain then recruited the death domain associated proteins that binds to caspase-8. P62 could be bind to polyubiquitinated casp-8 and recruits casp-8 to the autophagosome membranes. In heart illnesses such dilated cardiomyopathy, myocardial infarction, and left ventricular pressure overload, circulating and cardiac TNF- levels are high. As a result, TNF- has been linked to the development of ventricular remodeling and cardiac dysfunction in infarcted hearts [44, 46].

## Conclusion

TRZ therapy caused cardiotoxicity in the current investigation, as evidenced by elevated cardiac enzymatic indices and significant histological alterations such as distributed chronic inflammatory cells with focal fragmentation of myocardial fibers and nuclei loss. TRZ's cardiotoxicity was caused by an increase in oxidative stress, inflammation, and autophagy. The antioxidant and scavenging properties of l-carnitine, on the other hand, helped to ameliorate these effects.

## Limitation

Further studies will be required to explore detailed mechanisms of cardio- protective effects produced by l-carnitine.

## Data Availability

Applicable on request.
